# Retention in care of infants diagnosed with HIV at birth: Beyond the diagnostic strategy

**DOI:** 10.4102/sajid.v39i1.589

**Published:** 2024-03-30

**Authors:** Michael J. Christie, Nicolette M. du Plessis

**Affiliations:** 1 Department of Paediatrics and Child Health, Faculty of Health Sciences, University of Pretoria, Pretoria, South Africa

**Keywords:** HIV, infant, point-of-care, loss to follow-up, diagnostics

## Abstract

**Background:**

Birth HIV point-of-care (POC) tests curtail analytical testing issues and expedite diagnosis, potentially allowing for earlier mother-infant pair engagement and improved outcomes. Many children are lost post antiretroviral therapy (ART) initiation within the first 6 months of follow-up.

**Objectives:**

We compared 6-month retention in care, HIV viral load (VL) suppression and mortality among infants diagnosed with HIV at birth, using laboratory-based versus POC HIV PCR testing.

**Method:**

From 2018 to 2019, infants exposed to HIV underwent birth HIV PCR POC testing at Kalafong Provincial Tertiary Hospital in Tshwane District. Their outcomes were compared to a historical control born between 2014 and 2016, who exclusively underwent laboratory-based HIV PCR testing. Both groups received comparable HIV care following national guidelines.

**Results:**

Fifty-seven infants were studied (POC: 27; Control: 30). The POC turnaround time was significantly shorter (POC: 15.5 h [IQR: 4.3–24.7], Control: 68.3 h [IQR 46.0–93.9]; *p* = < 0.0001). Both populations had the same elapsed time from HIV diagnosis to ART initiation (median: 13 days, POC: IQR 8–21 days; Control: IQR 9–36 days). Six infants were never initiated (POC: 2 [7%]; Control: 4 [13%]). At 6 months, overall care retention was 72% (41/57), higher among the Control group (Control 23/30, 77%; POC: 18/27, 67%). HIV viral suppression at 6 months was higher among the POC group (POC: 14/18, 78%; Control: 9/19, 47%, *p* = 0.09). No deaths were reported.

**Conclusion:**

Poor care retention at 6 months post ART initiation is concerning. Initial mother-infant visits should be effectively utilised to assess and manage potential risk factors for loss of follow-up.

**Contribution:**

This study highlights the ongoing need to find workable solutions to improve retention in care, thereby ensuring the benefits of expedited HIV diagnosis and ART initiation.

## Introduction

Expeditious HIV diagnosis and antiretroviral therapy (ART) initiation are crucial in managing children living with HIV (CLHIV). Mortality rates in untreated infants with perinatal HIV peak at 2–3 months of age, reaching 35% at 12 months.^1,2^ If ART is initiated before 12 weeks of age, mortality is reduced.^3^ Prompt ART initiation also reduces HIV viral reservoir size, improves virological control, immunological function and growth.^4,5,6,7,8,9,10,11^ Since 2010, the South African National Department of Health (NDoH) HIV guidelines evolved to incorporate this body of evidence. Guidelines recommended lifelong ART for all children living with HIV <1 year of age and pregnant and breast-feeding women living with HIV (so-called ‘Option B+’) in 2010 and 2013, respectively.^12,13^ In 2015, updated guidelines recommended universal HIV polymerase chain reaction (PCR) testing at birth and 10 weeks for all HIV-exposed infants at birth.^14^ This was a change from the previous 2013 guidelines of testing at 6 weeks.^13^ Consequently, the Early Infant Diagnosis (EID) programme led to improved testing coverage and earlier infant diagnosis.^15,16^

In 2017, the Joint United Nations Programme on HIV/AIDS (UNAIDS) set out the 90-90-90 targets, stating that by 2020 90% of all people would know their HIV status, of these 90% would be on ART, of whom 90% would be virally suppressed (i.e.: 90-90-90).^17^ By 2018, the South African EID coverage was at 88.7% with 14 000 children (0–14 years) newly diagnosed with HIV. HIV was confirmed in 76% of children living with HIV, of whom 63% were on ART with 46% being virally suppressed.^18^

Globally, the standard of care for diagnosing HIV in infants is laboratory-based HIV PCR tests. However, to curtail laboratory-based pre- and post-analytical testing issues, HIV PCR Point-of-Care tests (POCT) were developed, validated,^19,20,21,22^ and shown to be beneficial in reducing cost, and result turn-around time (TAT) (<24 hours), and improving time to ART initiation and life expectancy.^23,24,25^

Regardless of the testing modality, success of an HIV programme depends on retaining patients in care.^26^ Keeping children living with HIV in care remains a global challenge: 5% – 29% of children living with HIV are not retained in care by 12 months post ART initiation, with most lost within the first 6 months of follow-up.^27,28^ This study aimed to describe retention in care rates at age 6-months of two historical infant cohorts after HIV birth diagnosis using two EID testing modalities: the first using POCT and the second using a centralised laboratory-based testing. We hypothesised that POCT would allow for earlier engagement with the mother–infant pair and improve retention in care.

## Materials and methods

### Study design

A secondary, descriptive analysis was done using data from two previous studies conducted at Kalafong Provincial Tertiary Hospital (KPTH) in Tshwane District, South Africa.^29,30^

The point-of-care (POC) group formed part of a prospective, implementation study for EID POC HIV PCR testing. Infants exposed to HIV at birth were enrolled between 01 July 2018 and 30 June 2019 and received POC HIV PCR testing. Infants with positive POC HIV PCR test results received an additional confirmatory POC HIV PCR and laboratory-based HIV PCR test and were managed by the paediatric immunology clinic at KPTH.^30^ The control group formed part of a study to assess the feasibility of using models for targeted birth HIV PCR testing in infants at high risk for HIV vertical transmission, between 01 August 2014 and 31 December 2016. Infants with positive HIV PCR results received confirmatory laboratory-based HIV PCR tests and were managed at the same clinic.^29^

The following outcomes were compared: result return before discharge, TAT and agreement between POC results and laboratory results.

The POC HIV PCR test utilised whole blood sampled within 72-h post-delivery and tested on-site using the Cepheid Xpert^®^ HIV-1 Qualitative assay (Cepheid, Sunnyvale, CA). Laboratory-based HIV PCR tests were conducted using whole blood sampled within 72-h post-delivery and tested at the National Health Laboratory Service (NHLS) laboratory using COBAS^®^ TaqMan^®^ HIV-1 Qualitative Test Version 2·0 (Roche Molecular Systems, Inc., Branchburg, NJ). Confirmatory laboratory-based HIV PCR testing was performed for all neonates, and an HIV-positive status was assigned to neonates with an ‘HIV-detected’ confirmatory result.

### Study setting

In 2017, an estimated 6850 children living with HIV resided within Tshwane District; of whom 52% were receiving ART.^31,32^ At KPTH, children living with HIV are managed at the on-site, multi-disciplinary paediatric immunology clinic. The clinic is staffed by nurses and doctors and assisted by dieticians, occupational therapists and social workers. It is overseen by two paediatric infectious disease specialists.

### Study population

All infants born at KPTH and diagnosed with HIV through either laboratory-based or POC HIV PCR tests, irrespective of gestational age, comorbid conditions and birth weight, were included in the analysis. Infants not receiving a POCT or laboratory-based HIV PCR test ≤ 72 h of life were excluded. Infants kept in hospital were classified as either asymptomatic or symptomatic.

### Definitions

Symptomatic infants were defined as requiring any medical care and intervention. Asymptomatic infants were clinically well, did not require any medical care or intervention and were only kept in hospital for social reasons. Antiretroviral initiation was considered delayed if initiated > 7 days after diagnosis. Loss to follow-up (LTFU) was loss of patients from care at KPTH, at any point during the study period, for >90 days after the last scheduled appointment.^33^ HIV viral suppression was defined as a viral load (VL) below detectable limits (lower than detectable [LDL]).^13,14^ All patients at 6-months of age who were still in care at KPTH or had a 6-month HIV VL result available on the NHLS database were defined as retained in care. For patients no longer in care at KPTH, the NHLS TrakCare database was used to search for HIV VL results during the follow-up period; this served as a proxy for evidence of contact at healthcare facilities other than KPTH. The interval used in this study to define the 6-month HIV VL was any HIV VL test done between 5 and 7 months of age.

Both groups were managed in accordance with NDoH guidelines in which all children < 5 years were eligible for ART.^13,14,34^ Follow-up occurred at monthly intervals.

### Data analysis

Collected data were exported to an Excel 365 workbook (Microsoft, USA) for analysis. The following maternal parameters were recorded: maternal age, nationality, duration of ART, maternal HIV VL result at delivery (HIV-1 RNA copies/mL). Infant parameters at birth included gender, gestational age, birth weight, baseline CD4 percentage, time elapsed from diagnosis to ART initiation, duration of hospital stay and reasons thereof. Six-month infant outcomes were LTFU, HIV-related mortality, retention in care and HIV VL suppression. At 6 months, the proportion of live infants in the two groups in care at KPTH or had an HIV VL test result available on the NHLS TrakCare database were compared. Descriptive statistics were used to describe baseline characteristics. Categorical data were summarised by using proportions. Median and inter-quartile range were used for skewed variables. Chi-square, fisher exact tests and Wilcoxon signed-rank test were used to test significance.

### Ethical considerations

The University of the Witwatersrand Human Subjects Research Ethics Committee (M1711115) and the University of Pretoria Research Ethics Committee (285/2014, 50/2018) approved the primary studies.^29,30^ Ethical approval was obtained for this study from the University of Pretoria Research Ethics Committee (244/2019). Approval was obtained from KPTH management to access the patient files and collected data were anonymised.

## Results

**FIGURE 1:**
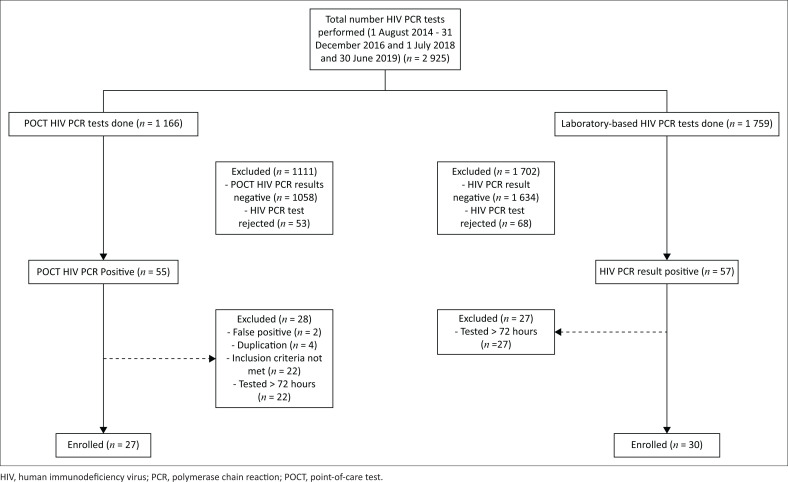
Consolidated consort diagram for the POCT group study period from 01 July 2018 to 30 June 2019 and the Control group study period from 01 August 2014 to 31 December 2016.

A total of 1166 HIV PCR POC tests were performed from 01 July 2018 to 30 June 2019. Of these, 55 tests (4.7%) were positive, 28 patients were excluded, and 27 neonates were subsequently enrolled. In the historical control group, 1759 infants with laboratory-based HIV PCR tests, 57 infants (3.2%) were positive, with 30 infants meeting the inclusion criteria. In both groups, delayed testing (>72 h of age) was the main reason for exclusion (Figure 1).

### Neonate characteristics

Compared to the historical control group, infants within the POC group tended to be more premature (median gestation 36 weeks versus 40 weeks, *p* = 0.06), and hospitalised (19/27, 70% vs. 15/30, 50%, *p* = 0.18). Median birth weights in both groups were similar (POC: 2 585g [interquartile range {IQR}: 2325 g – 3335 g], Control: 2700 g [IQR: 2280g – 3100g]). Of the 19 POC infants kept in hospital, 9 infants (9/19, 47%) were asymptomatic and kept for maternal pre-ART counselling and ART initiation, and 10 infants (10/19, 53%) were symptomatic. Fifteen infants in the control group were kept in hospital, 10 infants were symptomatic (10/15, 67%) and five infants (5/15, 33%) were asymptomatic and kept for either ART initiation (*n* = 4/15 27%) or maternal reasons (*n* = 1). Reported TAT in the POC group was significantly lower than the control group (POC: 15.5 h [IQR: 4.3–24.7 h], Control: 68.3 h [IQR 46.0–93.9 h]; p ≤ 0.0001). The median time from HIV diagnosis to ART initiation was similar in both groups (13 days). Six infants were never initiated (POC: 2/27, 7%; Control: 4/30, 13%) (Table 1).

**TABLE 1:** Baseline characteristics of Mother–Infant Pair at delivery at Kalafong Provincial Tertiary hospital between 01 July 2018 and 30 June 2019 (POCT group) and 01 August 2014 and 31 December 2016 (Control group).

Variable	POCT group (*n* = 27)				Control group (*n* = 30)				*p* †
	*n*	%	Median	IQR	*n*	%	Median	IQR	
**Infant characteristics**									
**Child gender**									
Male	17	63	-	-	17	57	-	-	0.78
Female	10	37	-	-	13	43	-	-	-
**Gestation (weeks)‡**									
Term (≥ 37)	14	56	36	35–40	24	86	40	37–40	0.06
Preterm (< 37)	11	44	-	-	4	14	-	-	-
**Birth weight (grams)‡**									
< 2500	9	35	2585	2325–3335	8	31	2700	2280–3100	0.42
≥ 2500	17	65	-	-	18	69	-	-	-
Reported TAT for HIV test results (hours)	-	-	15.5	4.3–24.7	-	-	68.3	46.0–93.9	< 0.0001¶
**Time elapsed from diagnosis to initiation (days)**									
≤ 7	4	15	13	8–21	4	13	12.5	9–36	0.83
8 – 28	16	59	-	-	15	50	-	-	-
> 28	5	19	-	-	7	24	-	-	-
Never initiated	2	7	-	-	4	13	-	-	-
**Baseline CD4 (%)‡**									
< 15	1	5	34.7	27.0–48.9	1	4	38.7	25.3–52.0	0.99
≥ 15	19	95	-	-	25	96	-	-	-
**Infant kept in hospital**									
Yes	19	70	-	-	15	50	-	-	0.18
No	8	30	-	-	15	50	-	-	-
**Maternal characteristics**									
Age (years)‡			29	26–33			28	23–33	-
≤ 18	2	9	-	-	3	12	-	-	0.93
> 18	21	91	-	-	23	88	-	-	-
Citizenship‡				-	-					
RSA citizen	12	52	-	-	18	72	-	-	0.23
Non-RSA citizen	11	48	-	-	7	28	-	-	-
ART duration‡				-	-					
< 3 months	9	39	-	-	10	34	-	-	0.78
≥ 3 months	14	61	-	-	19	66	-	-	-
**Maternal HIV VL at delivery (HIV ‑ 1 RNA copies/mL)‡**									
log(copies/mL)									
< 1000	5	25	4.37	3.3–5.02	5	19	4.1	3.61–4.85	0.72
≥ 1000	15	75	-	-	22	81	-	-	-

POCT, point-of-care test; IQR, interquartile range; CD4, cluster of differentiation; RSA, Republic of South Africa; ART, antiretroviral therapy; HIV, human immunodeficiency virus; VL, viral load; RNA, ribonucleic acid; TAT, turnaround time.

† , Chi square/Fischer’s exact test utilised unless otherwise specified.

‡ , Percentages and p-values were calculated using the known data; missing data was omitted from the calculations.

¶ , Wllcoxon Signed-Rank Test.

Significant difference shown if *p* < 0.05.

The documented reasons for delayed initiation (>7 days) in the POC group were indeterminant HIV PCR result (1/27, 4%), pre-ART counselling (7/27, 26%), concomitant disease (6/27, 22%) and LTFU after being discharged post-delivery (3/27, 11%). The documented reasons for delayed ART initiation within the control group were prolonged result TAT (12/30, 40%), indeterminate HIV PCR results (2/30, 6.7%) and concomitant disease (3/30, 10%).

### Maternal characteristics

Maternal ART duration was mostly ≥3 months (POC: 14/27, 52%; Control: 19/30, 66%); however, most mothers had an unsuppressed HIV VL result at delivery (POC: 15/27, 55%; Control 22/30, 73%) (Table 1).

### Follow up data

Overall retention in care at 6 months was 72% (41/57); 22 patients were LTFU at some point during the study period (POC: 11/27, 41%; Control: 11/30, 37%). By 6 months, more patients within the control group remained in care (Control: 23/30, 77%; POC: 18/27, 67%; *p* = 0.55) (Table 2). Twelve of the initially hospitalised infants were LTFU (POC: 6; Control: 6). HIV viral suppression at 6 months tended to be higher among the POC group (POC: 14/18, 78%; Control: 9/19, 47%, *p* = 0.09) (Table 2). No known deaths occurred over the study period in both groups; however, 16 patients (POC: 9; Control: 7) never returned to care (Table 2).

**TABLE 2:** Outcomes of the point-of-care test group and control group at 6 months of age.

Outcome	Total		POCT group		Control group		*p* †
	*n*	%	*n*	%	*n*	%	
**HIV positive at enrolment**	57	-	27	-	30	-	-
Lost to follow-up‡	22/57	39	11	41	11	37	0.79
Returned to care	6/22	27	2/11	18	4/11	36	0.63
Never returned to care	16/22	73	9/11	82	7/11	64	
**Retained in care at 6 months¶**	41/57	72	18/27	67	23/30	77	0.55
**HIV viral load available at 6 months (copies/ml)**	37	-	18	-	19	-	-
< 1000	23/37	62	14/18	78	9/19	47	0.09
≥ 1000	14/37	38	4/18	22	10/19	53	-
**Recorded mortality**	0	-	0	-	0	-	-

POCT, point-of-care test; HIV, human immunodeficiency virus; ml, millilitre; KPTH, Kalafong Provincial Tertiary Hospital; NHLS, National Health Laboratory Service.

‡ , Lost to follow-up was defined as loss of patient from care at KPTH, at any point during the study period, for >90 days after the last scheduled appointment.

¶ , Retained in care at 6 months defined as in care at KPTH or those who had a 6-month HIV viral load result available on the NHLS database.

† , Fischer’s exact test, significant difference shown if *p* < 0.05.

## Discussion

Early diagnosis is a crucial step in the care cascade of infants with perinatal HIV. Expedited ART initiation reduces mortality, lessens HIV viral reservoirs, improves growth parameters and reduces neurodevelopmental compromise and hospitalisation.^3,4,5,7,10,35,36^ Although the sample size was small, in this secondary analysis, POCT significantly reduced TAT (*p* ≤ 0.0001). Between the two groups, the time elapsed from diagnosis to ART initiation and the number of infants kept in hospital were not statistically different, while in hospital the reduced TAT created an opportunity for maternal pre-ART counselling and support prior to infant ART initiation. However, this counselling opportunity did not translate into improved retention at 6 months: retention in care remained poor irrespective of the testing modality. Retention in care rates for both groups were comparable to previously reported rates of approximately 70%.^28,37^

Our study was conducted in an academic hospital with admission capacity for mothers and infant pairs, thus ensuring early linkage to care and pre-ART counselling and support. This contrasts with many South African maternal obstetric units (MOU), situated in peripheral areas, where most deliveries occur. At these facilities, mothers are discharged after 6 h post normal vaginal delivery,^38^ and resources are unavailable to retain the mother–infant pair beyond the stipulated 6 h. Although POC is effective in peripheral settings to reduce TAT and improve ART initiation,^39^ children living with HIV in these areas are at a higher risk of LTFU.^40^ Purported risk factors for LTFU include caregiver (poor living conditions, lack of transportation, low caregiver education levels, social stigma, HIV status denial, seeking alternate faith-based or traditional treatment) and health system-related (fragmented HIV services, poor healthcare worker knowledge and support, lack of medication, waiting times, clinic distances) issues.^26,27,40,41^

Despite ART delayed initiation for some POC infants (>7 days) because of maternal pre-ART counselling (7/27, 26%), POC HIV PCR tests still proved marginally beneficial in improving ART initiation rates (POC: 93% vs. Control: 87%) and time from HIV diagnosis to ART initiation – most patients were initiated within 28 days (POC: 74% vs. Control: 63%). This benefit was observed in similar African studies.^25,42^ This study does highlight that if EID HIV diagnosis occurs in isolation, we will struggle to achieve the updated UNAIDS goals of 95-95-95. The first 6 months after diagnosing HIV is a particularly vulnerable period and action should be taken early to ensure patients are retained in care and offered adequate support. Every mother–infant pair living with HIV should be categorised as high risk and managed accordingly. Any healthcare contact, particularly the initial visits, should be utilised to assess for any barriers to adherence (caregiver and health system related), to offer counselling and support and to address any maternal needs.^27,43^ The family-centred care model can improve retention by providing coordinated care that addresses the health and social needs of all members of the family.^44^ Improved linkage to integrated HIV services after diagnosis, capturing correct patient contact details, telephonic appointment reminders, improved identification of patients who have missed appointments and better referral and case-finding procedures can further aid in improving retention in care.^26,27,28^ Continued research is warranted to identify factors to improve care retention and ART compliance in different settings.

This study has several limitations. The small cohort enrolled limited the study’s power to provide statistically significant outcomes. As utilised data came from different time periods, the variations between the study population’s baseline characteristics and management may have introduced biases. The low number of infants diagnosed with HIV bears witness to the ongoing effectiveness of the vertical transmission prevention programme. The group of patients initially categorised as LTFU and never returned to care underscores important considerations in accurately determining retention: results may not have been correctly retrieved as no unique national health-system identifier exists; patients may be enrolled in care at another facility under an alternate name and may also have been misclassified as LTFU rather than deceased if unrecorded deaths occurred. The latter is concerning: no deaths were reported in the study despite high infant mortality rates in the first few months of life among untreated infants living with HIV.^1,2^

## Conclusion

Retention rates of children living with HIV after ART initiation are an ongoing concern in our community and outweighs EID modality in our setting. As most patients are LTFU within the first 6-month post ART initiation, the initial visits should be effectively utilised to manage potential risk factors for LTFU. If workable solutions to improve retention are not implemented, possible gains of expedited diagnosis and ART initiation will be diminished. Ongoing research is warranted to determine factors at primary healthcare level to improve retention in care.

## References

[1] Violari A, Paed FC, Cotton MF, Babiker AG, Jean-Philippe P. Early antiretroviral therapy and mortality among HIV-infected infants. N Engl J Med. 2008;359:2223–2224. 10.1056/NEJMoa0800971 PMC2950021 19020325

[2] Bourne DE, Thompson M, Brody LL, et al. Emergence of a peak in early infant mortality due to HIV/AIDS in South Africa. AIDS. 2009;23(1):101–106. 10.1097/QAD.0b013e32831c54bd 19065753

[3] Marston M, Becquet R, Zaba B, et al. Net survival of perinatally and postnatally HIV-infected children: A pooled analysis of individual data from sub-Saharan Africa. Int J Epidemiol. 2011;40(2):385–396. 10.1093/ije/dyq255 21247884 PMC3140269

[4] Shiau S, Abrams EJ, Arpadi SM, Kuhn L. Early antiretroviral therapy in HIV-infected infants: Can it lead to HIV remission? Lancet HIV. 2018;5(5):e250–e588. 10.1016/S2352-3018(18)30012-2 29739699 PMC7487171

[5] Shiau S, Arpadi S, Strehlau R, et al. Initiation of antiretroviral therapy before 6 months of age is associated with faster growth recovery in South African children perinatally infected with human immunodeficiency virus. J Pediatr. 2013;162(6):1138–1145.e2. 10.1016/j.jpeds.2012.11.025 23312691 PMC3640753

[6] Kuhn L, Paximadis M, Da Costa Dias B, et al. Age at antiretroviral therapy initiation and cell-associated HIV-1 DNA levels in HIV-1-infected children. Sluis-Cremer N, editor. PLoS One. 2018;13(4):e0195514. 10.1371/journal.pone.0195514 29649264 PMC5896970

[7] Garcia-Broncano P, Maddali S, Einkauf KB, et al. Early antiretroviral therapy in neonates with HIV-1 infection restricts viral reservoir size and induces a distinct innate immune profile. Sci Transl Med. 2019;11(520):eaax7350. 10.1126/scitranslmed.aax7350 31776292 PMC8397898

[8] Massanella M, Puthanakit T, Leyre L, et al. Continuous prophylactic antiretrovirals/antiretroviral therapy since birth reduces seeding and persistence of the viral reservoir in children vertically infected with human immunodeficiency virus. Clin Infect Dis. 2021;73(3):427–438. 10.1093/cid/ciaa718 32504081 PMC8326541

[9] Goetghebuer T, Le Chenadec J, Haelterman E, et al. Short- and long-term immunological and virological outcome in HIV-infected infants according to the age at antiretroviral treatment initiation. Clin Infect Dis. 2012;54(6):878–881. 10.1093/cid/cir950 22198788

[10] Iyun V, Technau KG, Eley B, et al. Earlier antiretroviral therapy initiation and decreasing mortality among HIV-infected infants initiating antiretroviral therapy within 3 months of age in South Africa, 2006–2017. Pediatr Infect Dis J. 2020;39(2):127–133. 10.1097/INF.0000000000002516 31725119 PMC7073445

[11] Veldsman KA, Rensburg A, Isaacs S, et al. HIV-1 DNA decay is faster in children who initiate ART shortly after birth than later. J Intern AIDS Soc. 2019;22(8):e25368. 10.1002/jia2.25368 PMC6868516 31441231

[12] National Department of Health, Republic of South Africa. The South African antiretroviral treatment guidelines 2010. Pretoria: South African National Department of Health; 2010.

[13] National Department of Health, Republic of South Africa. The South African antiretroviral treatment guidelines. Pretoria: National Department of Health; 2013.

[14] National Department of Health, Republic of South Africa. National consolidated guidelines for the prevention of mother-to-child transmission of HIV (PMTCT) and the management of HIV in children, adolescents and Adults. Pretoria: National Department of Health; 2015.

[15] Cotton MF, Violari A, Otwombe K, et al. Early time-limited antiretroviral therapy versus deferred therapy in South African infants infected with HIV: Results from the children with HIV early antiretroviral (CHER) randomised trial. Lancet. 2013;382(9904):1555–1563. 10.1016/S0140-6736(13)61409-9 24209829 PMC4104982

[16] Innes S, Lazarus E, Otwombe K, et al. Early severe HIV disease precedes early antiretroviral therapy in infants: Are we too late? J Int AIDS Soc. 2014;17(1):18914. 10.7448/IAS.17.1.18914 24925044 PMC4056161

[17] Joint United Nations Programme on HIV/AIDS (UNAIDS). Ending AIDS Progress towards the 90–90-90 targets. [homepage on the Internet]. Joint United Nations Programme on HIV/AIDS (UNAIDS); 2017 [cited 2023 Mar 03]. Available from: https://www.unaids.org/en/resources/documents/2017/90-90-90

[18] Joint United Nations Programme on HIV/AIDS (UNAIDS). UNAIDS data 2019. Geneva: UNAIDS; 2019. 12349391

[19] Essajee S, Vojnov L, Penazzato M, et al. Reducing mortality in HIV-infected infants and achieving the 90-90-90 target through innovative diagnosis approaches. J Int AIDS Soc. 2015;18(7S6):20299. 10.7448/IAS.18.7.20299 26639120 PMC4670838

[20] Technau KG, Kuhn L, Coovadia A, Murnane PM, Sherman G. Xpert HIV-1 point-of-care test for neonatal diagnosis of HIV in the birth testing programme of a maternity hospital: A field evaluation study. Lancet HIV. 2017;4(10):e442–e448. 10.1016/S2352-3018(17)30097-8 28711526 PMC5623143

[21] Murray TY, Sherman GG, Nakwa F, et al. Field evaluation of performance of Alere and Cepheid qualitative HIV assays for pediatric point-of-care testing in an academic hospital in Soweto, South Africa. Caliendo AM, editor. J Clin Microbiol. 2017;55(11):3227–3235. 10.1128/JCM.01021-17 28855305 PMC5654906

[22] World Health Organization. WHO prequalification of in vitro diagnostics public report. Product: AlereTM q HIV-1/2 detect. WHO Reference Number PQDx 0226-032-00.57 [homepage on the Internet]. Geneva: World Health Organization; 2016 [cited 2023 Mar 03]. Available from: http://www.who.int/diagnostics_laboratory/evaluations/pq-list/hiv-vrl/160613PQPublicReport_0226-032-00AlereHIVDetect_v2.pdf

[23] Jean-Philippe P, Spiegel H, Gnanashanmugam D, et al. HIV birth testing and linkage to care for HIV-infected infants. AIDS. 2017;31(13):1797–1807. 10.1097/QAD.0000000000001561 28590330

[24] Frank SC, Cohn J, Dunning L, et al. Clinical effect and cost-effectiveness of incorporation of point-of-care assays into early infant HIV diagnosis programmes in Zimbabwe: A modelling study. Lancet HIV. 2019;6(3):e182–e190. 10.1016/S2352-3018(18)30328-X 30737187 PMC6408227

[25] Mwenda R, Fong Y, Magombo T, et al. Significant patient impact observed upon implementation of point-of-care early infant diagnosis technologies in an observational study in Malawi. Clin Infect Dis. 2018;67(5):701–707. 10.1093/cid/ciy169 29490026 PMC6093992

[26] Machine EM, Gillespie SL, Homedes N, et al. Lost to follow-up: Failure to engage children in care in the first three months of diagnosis. AIDS Care. 2016;28(11):1402–1410. 10.1080/09540121.2016.1179714 27160542 PMC5070915

[27] Abuogi LL, Smith C, McFarland EJ. Retention of HIV-infected children in the first 12 months of anti-retroviral therapy and predictors of attrition in resource limited settings: A systematic review. Okulicz JF, editor. PLoS One. 2016;11(6):e0156506. 10.1371/journal.pone.0156506 27280404 PMC4900559

[28] Carlucci JG, Liu Y, Clouse K, Vermund SH. Attrition of HIV-positive children from HIV services in low and middle-income countries. AIDS. 2019;33(15):2375–2386. 10.1097/QAD.0000000000002366 31764102 PMC6905128

[29] Du Plessis NM, Muller CJB, Avenant T, Pepper MS, Goga AE. An early infant HIV risk score for targeted HIV testing at birth. Pediatrics. 2019;143(6):e20183834. 10.1542/peds.2018-3834 31101703

[30] Kufa T, Mazanderani AH, Sherman GG, et al. Point-of-care HIV maternal viral load and early infant diagnosis testing around time of delivery at tertiary obstetric units in South Africa: A prospective study of coverage, results return and turn-around times. J Intern AIDS Soc. 2020;23(4):e25487. 10.1002/jia2.25487 PMC7180267 32329186

[31] Eaton JW, Dwyer-Lindgren L, Gutreuter S, et al. Naomi: A new modelling tool for estimating HIV epidemic indicators at the district level in sub-Saharan Africa. J Int AIDS Soc. 2021;24(S5):e25788. 10.1002/jia2.25788 34546657 PMC8454682

[32] South Africa District HIV Estimates. South Africa District HIV Estimates [homepage on the Internet]. 2022 [cited 2023 Jul 01]. Available from: https://www.hivdata.org.za/

[33] World Health Organization. Retention in HIV programmes: Defining the challenges and identifying solutions: Meeting report, 13–15 September 2011. Geneva: WHO Press; 2012, p. 64.

[34] National Department of Health. Implementation of universal test and treat strategy for HIV positive patients and differentiated care for stable patients [homepage on the Internet]. Pretoria: South African National Department of Health; 2016 [cited 2023 May 24]. Available from: https://sahivsoc.org/Files/22%208%2016%20Circular%20UTT%20%20%20Decongestion%20CCMT%20Directorate.pdf

[35] Benki-Nugent S, Tamasha N, Mueni A, et al. Early antiretroviral therapy reduces severity but does not eliminate neurodevelopmental compromise in children with HIV. JAIDS J Acquir Immune Defic Syndr. 2023;93(1):7–14. 10.1097/QAI.0000000000003165 36693138 PMC10079595

[36] Anderson K, Iyun V, Eley BS, et al. Hospitalization among infants who initiate antiretroviral therapy before 3 months of age. AIDS. 2023;37(3):435–445. 10.1097/QAD.0000000000003422 36695356 PMC9881839

[37] Technau KG, Strehlau R, Patel F, et al. 12-month outcomes of HIV-infected infants identified at birth at one maternity site in Johannesburg, South Africa: An observational cohort study. Lancet HIV. 2018;5(12):e706–e714. 10.1016/S2352-3018(18)30251-0 30416043 PMC6336389

[38] National Department of Health. Guidelines for maternity care in South Africa: A manual for clinics, community health centres and district hospitals. 4th ed. National Department of Health, South Africa; 2016.

[39] Bianchi F, Cohn J, Sacks E, et al. Evaluation of a routine point-of-care intervention for early infant diagnosis of HIV: An observational study in eight African countries. Lancet HIV. 2019;6(6):e373–e381. 10.1016/S2352-3018(19)30033-5 30987937

[40] Hibstie YT, Kibret GD, Talie A, Temesgen B, Melkamu MW, Alebel A. Nearly one in every six HIV-infected children lost from ART follow-up at Debre Markos Referral Hospital, Northwest Ethiopia: A 14-year retrospective follow-up study. Marotta C, editor. PLoS One. 2020;15(9):e0239013. 10.1371/journal.pone.0239013 32931502 PMC7491726

[41] Mofenson LM, Cohn J, Sacks E. Challenges in the early infant HIV diagnosis and treatment cascade. JAIDS J Acquir Immune Defic Syndr. 2020;84(1):S1–S4. 10.1097/QAI.0000000000002366 32520908

[42] Luo R, Fong Y, Boeras D, Jani I, Vojnov L. The clinical effect of point-of-care HIV diagnosis in infants: A systematic review and meta-analysis. Lancet. 2022;400(10356):887–895. 10.1016/S0140-6736(22)01492-1 36116479

[43] National Department of Health. Adherence guidelines for HIV, TB and NCDs: Policy and service delivery guidelines for linkage to care, adherence and retention in care. National Department of Health, South Africa; 2016.

[44] Leeper SC, Montague BT, Friedman JF, Flanigan TP. Lessons learned from family-centred models of treatment for children living with HIV: Current approaches and future directions. J Int AIDS Soc. 2010;13:S3. 10.1186/1758-2652-13-S2-S3 PMC2890972 20573285

